# Single-Shot near Edge X-ray Fine Structure (NEXAFS) Spectroscopy Using a Laboratory Laser-Plasma Light Source

**DOI:** 10.3390/ma11081303

**Published:** 2018-07-28

**Authors:** Przemysław Wachulak, Martin Duda, Tomasz Fok, Andrzej Bartnik, Zhanshan Wang, Qiushi Huang, Antoni Sarzyński, Alexandr Jancarek, Henryk Fiedorowicz

**Affiliations:** 1 Institute of Optoelectronics, Military University of Technology, 2 Urbanowicza Str., 00-908 Warsaw, Poland; tfok@wat.edu.pl (T.F.); abartnik@wat.edu.pl (A.B.); asarzynski@wat.edu.pl (A.S.); hfiedorowicz@wat.edu.pl (H.F.); 2 Faculty of Nuclear Sciences and Physical Engineering, Czech Technical University in Prague, Břehová 7, 115 19 Praha 1 Prague, Czech Republic; dudamar1@fjfi.cvut.cz (M.D.); alexandr.jancarek@fjfi.cvut.cz (A.J.); 3 Key Laboratory of Microstructured Materials of MOE, School of Physics Science and Engineering, Tongji University, Shanghai 200092, China; wangzs@tongji.edu.cn (Z.W.); huangqs@tongji.edu.cn (Q.H.)

**Keywords:** NEXAFS, soft X-rays SXR, SXR absorption spectroscopy

## Abstract

We present a proof of principle experiment on single-shot near edge soft X-ray fine structure (NEXAFS) spectroscopy with the use of a laboratory laser-plasma light source. The source is based on a plasma created as a result of the interaction of a nanosecond laser pulse with a double stream gas puff target. The laser-plasma source was optimized for efficient soft X-ray (SXR) emission from the krypton/helium target in the wavelength range from 2 nm to 5 nm. This emission was used to acquire simultaneously emission and absorption spectra of soft X-ray light from the source and from the investigated sample using a grazing incidence grating spectrometer. NEXAFS measurements in a transmission mode revealed the spectral features near the carbon K-α absorption edge of thin polyethylene terephthalate (PET) film and L-ascorbic acid in a single-shot. From these features, the composition of the PET sample was successfully obtained. The NEXAFS spectrum of the L-ascorbic acid obtained in a single-shot exposure was also compared to the spectrum obtained a multi-shot exposure and to numerical simulations showing good agreement. In the paper, the detailed information about the source, the spectroscopy system, the absorption spectra measurements and the results of the studies are presented and discussed.

## 1. Introduction

X-ray absorption fine structure (XAFS) spectroscopy allows for the chemical environment of the sample to be investigated by studying X-ray absorption of the sample in the vicinity and above the core level binding energy of the considered atom. A modulation in the X-ray absorption spectrum of an atom carries information about its physical and chemical states. A near-edge X-ray absorption fine structure (NEXAFS) spectroscopy is typically performed in the energy range from a few eV below the absorption edge of the investigated atom to, typically, 20–30 eV above the absorption edge. It is a well-established technique for the characterization of chemical and environmental compounds [1], including organic materials (composed of carbon, oxygen, and nitrogen) that exhibit absorption edges in the soft X-ray (SXR) spectral region, λ = 0.1–10 nm wavelength. The SXR NEXAFS yields information about elemental composition through the observation of the spectral features in the vicinity of the X-ray absorption edge [2], for studies of the intermolecular bond structure of polymers [3] and saccharides [4], or for obtaining polymer fingerprint of the material [5]. NEXAFS was also used to study the functions of low dimensional nanostructures [6], to investigate liquids [7] and nanomaterials [8], or probing electronic and chemical states of battery materials [7,8].

NEXAFS spectroscopic investigations are usually performed using synchrotron radiation (SR) facilities, which provide radiation of very high brilliance and intensity over a broad wavelength/energy range in the X-ray spectral region. However, for SR NEXAFS spectrum acquisition a wavelength//energy scanning approach is used, which is time-consuming and does not allow for time-resolved studies. A concise overview of this can be found in the article, in which motivation for an easy accessed complimentary NEXAFS technique based on a compact X-ray source was pointed out [9]. Several NEXAFS systems, operating in the SXR range, have been developed using laboratory laser plasma light sources driven with picosecond and nanosecond lasers [10,11,12,13]. These systems were used in the studies of various materials in vacuum [11,12,13], while the NEXAFS system based on the source driven with a picosecond laser was used in the investigations on photo induced phase transitions studies [14]. More recently, high order harmonic generation (HHG) sources were also used for soft X-ray spectroscopy [15] reaching even the “water window” spectral range (λ = 2.3–4.4 nm) [16]. Most of the studies, however, required relatively long, multi-pulse exposure to acquire a single NEXAFS spectrum [12], which may become an obstacle to investigating biological specimen or samples that change with time. An example of this is a single-gas jet laser-plasma SXR source employed for NEXAFS experiments [17], in which a very long exposure time, reaching up to ten thousand pulses [18], was necessary to reach sufficient signal to noise ratio to obtain a single NEXAFS spectrum. Such approach discards the possibility for high throughput measurements. To overcome this limitation, a single pulse (single-shot) has to be used for NEXAFS spectrum acquisition. A single-shot NEXAFS has been demonstrated recently using a laser plasma light source based on a solid target [19]. However, solid targets that are known to produce debris associated with laser ablation products, which is a highly undesirable effect. Moreover, the design of the spectrometer, including two separate off-axis zone plates, may be prone to mechanical, vibration instabilities, errors in the alignment of the sample and reference spectra for two spectra acquired separately and integration errors in the minute curvature of lines of equal energy in the spectra obtained using off-axis zone plates. 

In this paper, we demonstrate a single-shot NEXAFS experiment with the use of a laser plasma light source, based on a double stream gas puff target, which injects two gasses into the laser-matter interaction region, to improve the overall photon yield from such produced plasmas [20]. The target was irradiated with modest (a few joules) energies of the laser pulses. In the gas puff target, the inner gas was chosen for a specific elemental emission, while the outer gas that surrounds the inner gas decreases the density gradient of the inner gas in the direction of the nozzle axis. This significantly increases the target density in the interaction region and allows to obtain higher extreme ultraviolet (EUV) and SXR yields at more modest pumping conditions. Moreover, the gaseous target does not have a problem with a debris production.

Thus, in this work, we demonstrate single-shot NEXAFS measurements on the thin organic samples with the laser plasma SXR source employing a double stream gas puff target. The SXR emission from krypton/helium plasma, allowed one to perform NEXAFS with a 1.3 ns exposure time. As a proof of principle, a 1 μm thick polyethylene terephthalate (PET) and L-ascorbic acid samples were used. Optical density spectra of both samples were obtained with a single SXR pulse exposure and composition of the PET sample was evaluated to confirm the applicability of laser plasma source, based on a double stream gas puff target, to NEXAFS measurements, obtaining a useful single-shot signal.

As a result, a NEXAFS system was developed, based on 10 J, 1 ns Nd:YAG laser system. In this approach, a simultaneous acquisition of reference and sample spectra was possible, through a specially designed SXR spectrometer equipped with long entrance slit. Such construction facilitates the much more accurate acquisition of the spectra, which are independent of source energy fluctuations as well as mechanical instabilities of the system. The spectral resolution of this compact system is comparable with early synchrotron-based works. In the following sections, the details about this system will be presented and discussed.

## 2. Experimental Setup

**Figure 1:**
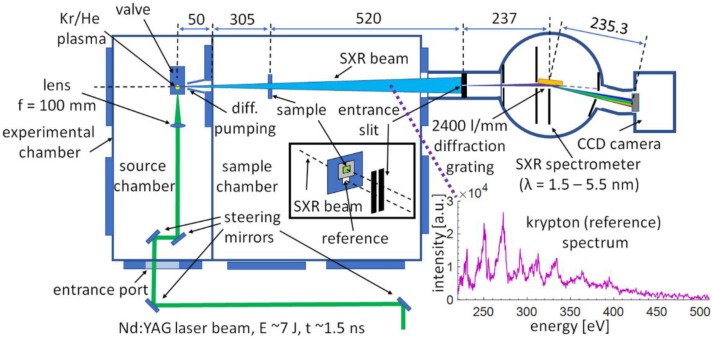
Optical arrangement for single soft X-ray (SXR) pulse experimental near edge soft X-ray fine structure (NEXAFS) system employing laser plasma source based on a double stream gas puff target.

**Figure 2:**
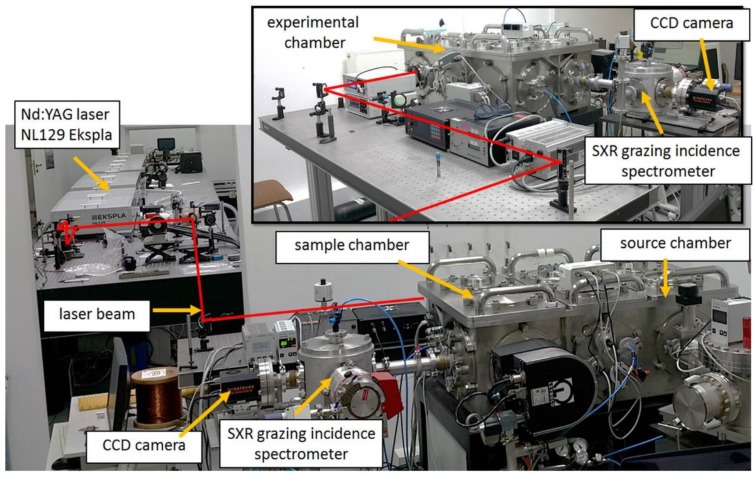
Photograph of the single SXR pulse NEXAFS system.

The experimental setup for the single SXR pulse NEXAFS system using the emission from krypton/helium plasma is depicted in Figure 1 and the photograph of the system is depicted in Figure 2.

An Nd:YAG laser beam, emitted from an NL 129 laser system, maximum energy 10 J, (EKSPLA, Vilnius, Lithuania), with laser pulse energy ranging from ~2 J to ~7 J, depending on the measurements, and ~1.5 ns time duration, is focused by an f = 10 cm focal length lens onto a double stream gas puff target. The target is produced by a collinear set of two nozzles, driven independently by two electromagnetic valves.

The diameters of the nozzles are 0.4 mm for the inner nozzle and 0.7–1.5 mm for the outer, ring-shaped nozzle. The inner nozzle was pressurized with krypton gas (working gas) at an optimum backing pressure of 11 bar, while the outer nozzle was connected to helium (outer gas) pressurized to 5 bar. The double stream gas puff target approach was used to increase the gas puff target density. It is done by injecting Kr gas into a hollow stream of He gas to shape the flow of the inner gas into a vacuum through the use of the outer gas. In such case higher inner gas density and SXR yield is obtained comparing to single stream gas puff approach [21]. The valves were driven separately by a dedicated two-channel controller, which is capable of independent adjustment of the delay and opening time for each valve. Moreover, driving signals for both valves are synchronized with the laser oscillator. 

Due to the interaction of the laser pulses with the gaseous target, a laser produced plasma is created. Such plasma emits radiation in the broad range of wavelengths, from soft X-rays to infrared, depending on the gas used as a target, laser beam and focusing system parameters. In this experiment, an efficient soft X-ray emission from krypton was achieved (as depicted in the inset of Figure 1, for E = 6.7 J laser pulse with 1.3 ns time duration) and employed for a single SXR pulse NEXAFS spectroscopy. The radiation from krypton plasma enters the second vacuum chamber (sample chamber, see Figure 1), where it illuminates the sample, which is being investigated, placed 355 mm from the plasma. The sample holder is designed in such a way to allow simultaneously for the SXR light to be transmitted through the sample (sample beam), but a portion of the SXR light (reference beam) also enters undisturbed the entrance slit of the spectrometer, see inset in Figure 1, located 875 mm from the plasma. Thus, in this system, in contrary to other compact systems [9] and similar to our recent measurements with the compact NEXAFS system [22,23], a simultaneous acquisition of the two spectra has been achieved. This solution has significant advantages. One of them is that the system remains unaffected by the energy fluctuations of the source, but it is also immune to mechanical instabilities of the system that may occur during a separate acquisition of sample and reference spectra. This, in turn, may lead to unpredicted spectral shifts and difficulty in calculating the optical density of the sample in the vicinity of the absorption edge. For simultaneous acquisition of two spectra the SXR beams enter the spectrometer through the elongated entrance slit, 15 mm in length, which width was 12 μm. The slit was fabricated in a 50 μm thick brass foil by repetitive ablation of the material due to interaction with a focused laser beam with a metal sheet.

The NEXAFS spectra are obtained using a home-made spectrograph with a grazing incidence diffraction grating from Hitachi High Technologies America Inc., Baltimore, MD, USA, having 2400 lines per mm, wavelength range from 1 nm to 5 nm, and a back-illuminated CCD camera (GE 20482048, greateyes GmbH, Berlin, Germany), placed downstream the diffraction grating, in configuration reported in [24]. The camera has a chip with 2052 × 2046 pixels, each 13 × 13 μm^2^ in size. During the experiments the chip was cooled down to −40 °C to reduce its internal noise and the background.

For the efficient emission in the SXR region used in the NEXAFS experiment, especially to be able to record high signal to noise ratio reference and sample spectra in a single SXR pulse, the krypton/helium target laser plasma source was properly optimized. The optimization concerned the gas puff target delays in respect to the synchronization pulse from the laser oscillator, arriving 1 ms before the laser pulse, yielded delay times for krypton equal to 500 μs and krypton valve opening time of 800 μs, while the same parameters for helium were 500 μs and 700 μs, respectively. More about synchronization and timing can be found in [22,25]. To avoid reabsorption of SXR radiation generated from laser-plasma, the laser focus was located not in the center of the nozzle but shifted in direction of the optical system by 0.5 mm. Moreover, the working and outer gas pressures were also optimized for maximum SXR photon yield from krypton/helium laser-plasma resulting in optimum pressure values of 11 bar for krypton and helium pressure of 5 bar. If the pressure of the gas is being increased, the density of the target increases as well, and so does the photon yield. After the optimum value of the pressure is reached, further increasing the backing pressure causes the target density to decrease. This is due to the fact that high backing pressure in the valve reservoir prohibits the valve to fully open, because of limited energy stored in the capacitor bank that is discharged through the valve coil while it operates.

The key part of the single SXR pulse NEXAFS system is a high spectral resolution grazing incidence SXR spectrometer. For its proper operation, a precise calibration of the spectrometer is essential because the spectral features near the absorption edge ought to be properly defined and distinguished with energy accuracy of a fraction of an eV. The laser plasma SXR source based on a double stream gas puff target allows one to perform such calibration easily because the change in spectral emission can be obtained by changing the inner, working gas. For that purpose, initially, three different gasses were used: argon, oxygen, and nitrogen. Those gasses have in their emission spectra single, well visible, and easy to recognize isolated emission lines [26]. 

**Figure 3:**
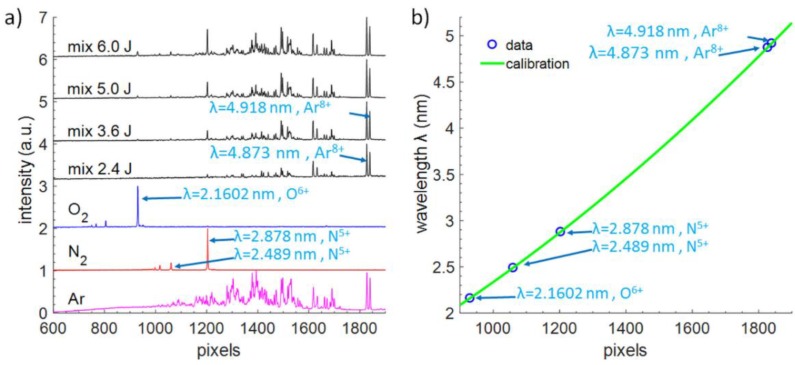
SXR spectrometer calibration. SXR spectra (**a**) of the pure gasses (three bottom plots) and a 1:1:1 (by volume) of Ar, N_2_, and O_2_ gasses for different energies of the laser pulses. A mixture (mix) is more convenient to perform calibration and allows one to see all lines of interest, later used to obtain a spectrometer calibration curve, depicted in Figure (**b**).

Such lines are shown in Figure 3a for those three gasses and their wavelength range spans from λ = 2.1602 nm line from O^6+^ ion in O_2_–based plasma, through two well-defined and most intense SXR nitrogen lines: λ = 2.489 nm and λ = 2.878 nm from N^5+^ ions, till λ = 4.873 and λ = 4.918 nm lines from Ar^8+^ ions. In this experiment, however, instead of three different gasses for spectrometer calibration we prepared and used an Ar:N_2_:O_2_ (1:1:1 by volume) gas mixture for easier, faster and more precise calibration of the spectrometer, since in one spectrum multiple transitions (spectral emission lines) from different gasses are present and could be used for the calibration process. Those lines were used to obtain the calibration curve for the spectrometer, Equation (1). To do that a parabolic function was fitted to the data with R-squared fitting equal to 0.(9)_6_648, (1 − R^2^ = 3.52·10^−7^), resulting in maximum wavelength error for 2.878 nm N^5+^ line equal to 0.017% and minimum error of −0.031% for 2.489 nm line from N^5+^ ions.

(1) $$y(x)=6.36\cdot 10^{-7}x^{2}+1.27\cdot 10^{-3}x+0.43$$

where *y* is the wavelength in nm, while the *x* value defines the pixel index in the CCD camera image, horizontal axes in Figure 3a,b.

The resolving power of the SXR spectrometer, equipped with 12 μm entrance slit, was estimated in the vicinity of the carbon absorption edge by measuring an isolated line at 4.409 nm wavelength (2s^2^2p^3^-2s^2^2p^2^(3P)3d transition from S^9+^ ions from SF_6_ gas, the photon energy of 281 eV). It was observed that the line has FWHM width of 0.3 eV, thus E/ΔE was estimated to be ~940. 

## 3. Experimental Results for a Single SXR Pulse near Edge X-ray Absorption Spectroscopy of PET and L-Ascorbic Acid

**Figure 4:**
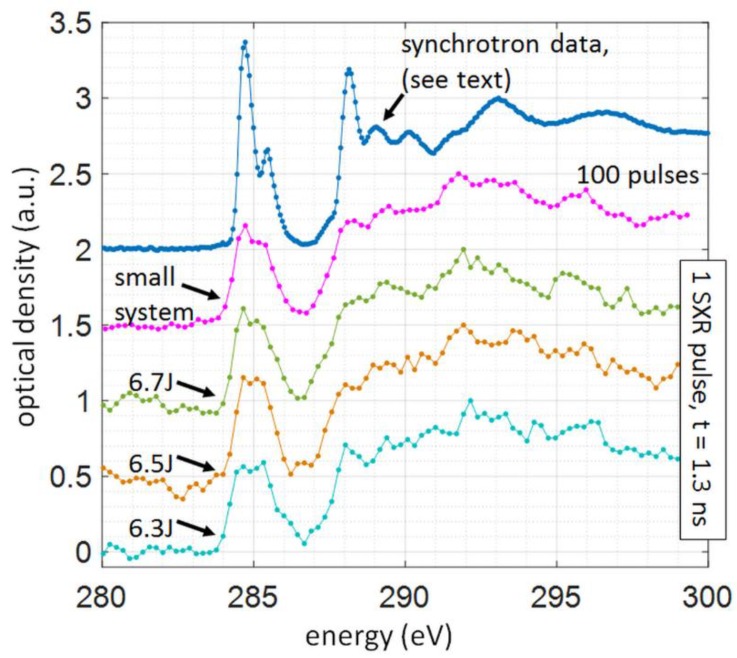
Polyethylene terephthalate (PET) NEXAFS spectra—optical density as a function of SXR radiation energy. The figure depicts a comparison between NEXAFS spectrum obtained using a synchrotron radiation [3], 100 SXR pulses from the compact system [22] with NEXAFS spectra of the same material and thickness, obtained with a single SXR pulse, generated by a laser interaction with a double stream gas puff target. The spectra are presented for three different laser pulse energies ranging from 6.3 J to 6.7 J and 1.3 ns laser pulse duration (FWHM).

To demonstrate the performance of the experimental system for a single SXR pulse NEXAFS spectroscopy a proof of principle experiment for obtaining NEXAFS spectra from 1 μm thick PET foil, (C_10_H_8_O_4_)_n_, (Lebow, USA) was performed. This experiment was already performed using the compact NEXAFS system, however, for single NEXAFS spectrum acquisition typically 100 SXR pulses were required [22]. In this experiment, the foil was partially covering the elongated aperture in the sample holder to allow two beams (sample and reference beams, indicated in the inset in Figure 1) to enter the spectrometer slit. Thus, the sample *S_sam_(E)* and reference spectra *S_ref_(E)* were acquired simultaneously with one SXR pulse from krypton/helium plasma. Typical optical density *OD* NEXAFS spectra for PET foil obtained with this system are depicted in Figure 4, for various laser energies ranging from 6.3 J to 6.7 J, and were obtained with Equation (2):

(2) $$OD(E)=-\ln\left[\frac{S_{sam}(E)}{S_{ref}(E)}\right].$$

The reference spectrum, which is the emission from Kr/He plasma, spans typically from 2.2 nm up to a detection limit of our grazing incidence spectrometer (GIS), which is 5 nm, corresponding to the energy of 225 eV to 560 eV. The sample spectrum shows clearly the carbon K-α absorption edge in the vicinity of 4.3 nm wavelength, above which the absorption of carbon is low. This results in a very well distinguishable and characteristic features in the NEXAFS spectrum, depicted in Figure 4. 

The NEXAFS spectrum is typically slightly smoothed out using the Golay-Savitzky algorithm, following [9], or by averaging the separately acquired spectra. A NEXAFS spectrum of PET, presented in Figure 4, was obtained by vertical integration of 101 spectral lines using a single data set and single laser pulse, 1.3 ns in duration, without any filtering procedure or multiple spectra accumulations. Similarly to compact system data (100 laser pulses with energies of 650 mJ and pulse duration 3 ns), reported in [22] (second-from-top plot in Figure 4), the most prominent feature of the spectrum is a π*_C=C_ bond from the aromatic ring in the PET structure at an energy of 284.4 eV and 285.1 eV. The other peaks in the spectrum were also identified and are listed in Table 1. The peaks were assigned based on the synchrotron data for poly(ethylene terephthalate) [27].

The spectral resolution of the NEXAFS data is sufficient to successfully fit two spectral contributions in a π*_C=C_ bond from the aromatic ring in the PET structure at photon energies of 284.4 eV and 285.1 eV, in accordance with synchrotron data [27] and similarly to synchrotron measurements [3] (top plot in Figure 4). Based on the resolving power of the spectrometer the spectral resolution of this single-shot NEXAFS system, ~0.3 eV, is comparable with early synchrotron-based works, however, more recent data [3] report better spectral resolutions of ~0.1 eV.

**Figure 5:**
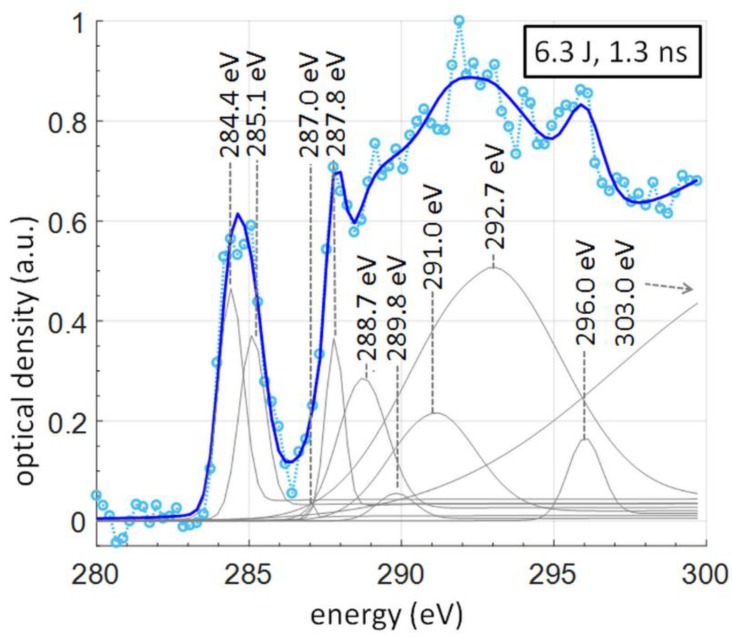
A NEXAFS spectrum of a 1 μm thick PET foil in the vicinity of carbon edge, obtained with one SXR pulse. Measured data points are indicated with circles, thick solid line depicts the fitting. Each contribution to the fitting is depicted by a thin solid line. A combined approach [9] was used, in which for each peak a separate 10% step function was employed. From the peaks composition of the PET sample was subsequently calculated.

For chemical composition analysis the peaks, corresponding to certain bonds in the molecular structure, as well as a step function, describing the profile of the absorption edge, are fitted, similarly to other works [9,19] and to our previous measurements with multiple SXR pulses and compact NEXAFS system [22]. The result of the fitting is depicted in Figure 5. For the step function, *arctan* was used, while for the peaks a pure Gaussian function was utilized [1]. To perform the peak fitting to the experimental NEXAFS spectral data a dedicated MATLAB software (R2018a, MathWorks, Natick, MA, USA) based on a nonlinear programming solver that searches for the minimum of an unconstrained multivariable function using the derivative-free method, was used. In the peak fitting algorithm, the only assumed parameters were the peak/step positions (energies) and the width of the step functions. All other parameters, such as all widths and amplitudes of the peak contributions (Gaussian functions) and heights of the step functions (based on the amplitudes of peak functions), were fitted automatically. 

During the data processing, we found that adding 10% *arctan* local step function to each spectral contribution described by a Gaussian peak function is sufficient to perform accurate fitting to the spectral data. This method, presented in [9], was used since it is not trivial to determine the exact position of a single, *arctan* global step function [1] for the measured data. The height of each step was set to 10%, because for the PET the ratio of absorption lengths around the carbon edge is equivalent to ~10%, according to the CXRO data [28]. 

### 3.1. Chemical Composition Analysis from a Single Pulse SXR PET NEXAFS Spectrum

For the elemental composition analysis, it was assumed that the area under each Gaussian peak curve assigned to certain resonance is proportional to the frequency of occurrence of this binding form of the studied element [29,30]. This allows one to approximate the composition of the bound elements. The spectral components, listed in Table 1, were fitted to the spectrum (thin gray curves under the dotted spectrum data points). The sum of all fitted curves is depicted as a blue line and it matches well the experimental data points. The energy positions and bond assignments were based on synchrotron data [27]. The spectral components listed in Table 1 are not the only ones that contribute, however, those are the most probable transitions, according to the literature, and were used for assignment of the peaks. 

For composition analysis, the sum of areas under each fitted spectral component was normalized to obtain a probability of occurrence of a particular bond. Not all spectral components from Table 1 were used for this normalization. The contribution of the σ* bonds has been accounted for from the π* orbitals, thus, components above 292 eV were left out of the estimation. Moreover, due to a disagreement in the assignment of the spectral component at 291 eV in the synchrotron data (Table 1 in [27], Okajima et al.) for PET, this contribution was also not accounted for. In different sources [27,31], the other spectral components are defined precisely with an energy accuracy of ~0.3–0.4 eV, while this particular spectral feature is assigned as carbonyl only in the data in [27] (Okajima et al.) while in the other reference it does not exist. Thus, since the existence and assignment of this component are not well established, we have decided to omit that contribution in the composition analysis. 

The results of composition evaluation for PET, compared to theoretical values, are presented in Table 2. For more information how the assignment was performed, based on the probability of occurring bonds please see [9,22]. By a comparison to the theoretical value, calculated as a percent by weight, *w/w* %, of the composition, the error of composition analysis was evaluated using a root-mean-square deviation approach defined by Equation (3):

(3) $$\delta =\frac{1}{N}\sqrt{\sum_{i=1}^{N}{\left(C_{Ti}-C_{Mi}\right)}^{2}}$$

where *N* is the number of elements, considered in the composition analysis (*N = 3*) and *i* defines the index for each element {C, H, O} = {1, 2, 3}. *C_Ti_* is a theoretical and *C_Mi_* is a measured percentage value for each element in the molecular structure.

From the error analysis, the global error was found to be ~2% for the composition analysis based on the experimental data. By value, the lowest error was found for hydrogen, equal to 0.4%, while the highest for carbon, equal to 5.1%. 

### 3.2. The NEXAFS Spectrum of L-Ascorbic Acid and Comparison to Numerical Simulations

**Figure 6:**
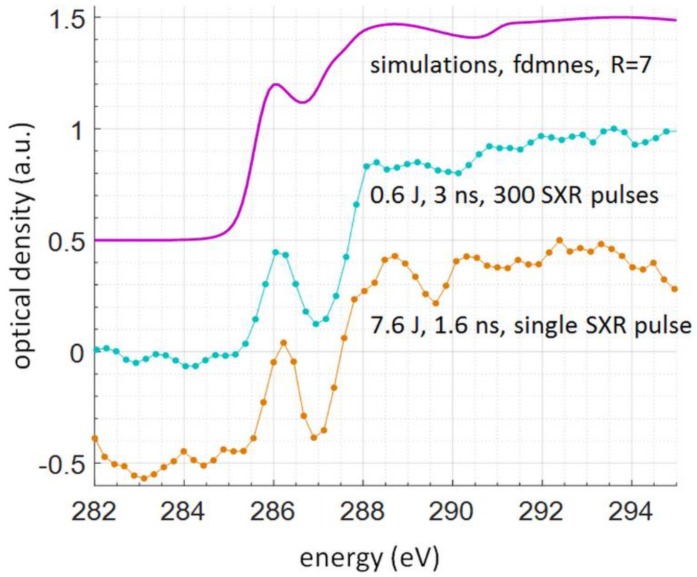
The relative values of the optical density of the L-ascorbic acid sample obtained near the carbon K-α absorption edge for multiple SXR pulses (compact system reported in [22]) and a single SXR pulse, compared to *fdmnes* numerical simulations (top plot).

Moreover, to validate the experimental NEXAFS spectra an organic sample of L-ascorbic acid, C_6_H_8_O_6_, was used. The L-ascorbic acid (99.9% purity, ~10 mg) in a powder form (Stanlab Sp.J., Lublin, Poland) was dissolved in distilled water (10 mL) using an ultrasonic cleaner. A 3 μL drop was then placed on top of 75 nm thick Si_3_N_4_ membrane acting as a support and dried in a nitrogen atmosphere for 5 min. The NEXAFS spectrum of L-ascorbic acid was obtained with a single SXR pulse, created by a 7.6 J and 1.6 ns in duration laser pulse. Such single-shot spectrum was compared with the one obtained with the compact NEXAFS system, in which NEXAFS spectrum using multiple SXR pulses was acquired. Moreover, both spectra were also compared directly with numerical simulations based on *fdmnes* software [32]. The comparison can be seen in Figure 6, where the correspondence between spectral features in both spectra is visible. The relative values of the optical density are chosen to separate the plots vertically.

The *fdmnes* simulations were performed with the region of interest of R = 7, and convolution parameter of 0.3 eV, based on lattice parameters and positions of atoms obtained from [33], with lattice parameters of a = 6.390(1) Å, b = 6.262(1) Å, c = 17.127(4) Å, α = γ = 90°, and β = 99.36°. The multiple SXR pulse NEXAFS spectrum was obtained using 300 SXR pulses emitted from the Kr/He plasma generated by the interaction of 0.6 J energy and 3 ns duration Nd:YAG laser pulses (laser beam diameter of ~7 mm), focused by an *f* = 25 mm lens. To obtain such spectrum 300 SXR pulses were necessary. A single SXR pulse NEXAFS spectrum was obtained using radiation from Kr/He plasma as well, however, the plasma was formed by the interaction of 7.6 J energy and 1.6 ns duration laser pulse (laser beam diameter of 25 mm and 100 mm focus lens) with a double stream Kr/He gas puff target. The focusing numerical apertures were similar, NA = 0.138 for the smaller system, comparing to NA = 0.124 for higher energy system. Thus, the beam diameters were also comparable. Even though the peak power for the latter case was 4.75 GW, comparing to 200 MW for the smaller laser, roughly 24 times smaller, the much more efficient Kr plasma emission allowed to obtain a NEXAFS spectrum for the same material of similar quality with just a single SXR pulse. 

## 4. Conclusions

In conclusion, the proof of principle experiment, showing the applicability of laser plasma source based on a double stream gas puff target to a single-shot NEXAFS, was demonstrated. The single-shot capability is important from the point of view of possible time-resolved studies. In this case, the time resolution of such studies is comparable to the duration of the single SXR pulse, 1.3 ns for PET and 1.6 ns for the L-ascorbic acid sample. This work demonstrates, that the laser-plasma source based on a double stream gas puff target can be employed for NEXAFS measurements in the laboratory environment, obtaining a useful single-shot signal. The table-top system based on this source was developed, and its application to study a polymer sample, showing its applicability to the near edge X-ray absorption fine structure spectroscopy, was demonstrated. The obtained PET spectrum is comparable in quality to the spectrum obtained with another compact desk-top system, requiring, however, 300 SXR pulses acquisition. The spectrum exhibits the expected peaks at the characteristic positions, which in turn allows performing the composition analysis, which results match the theoretical values of C, H, and O composition. Moreover, the NEXAFS spectrum of a real sample of L-ascorbic acid on top of the Si_3_N_4_ membrane also compares very well to multiple-shot data and to numerical simulations.

The system allows one to obtain the NEXAFS spectrum from the simultaneous acquisition of two spectra (sample and reference) with the exposure time of ~1 ns. Simultaneous acquisition of both sample and reference spectra makes possible for much more accurate data acquisition, independent of source energy fluctuations and mechanical instabilities of the system. For higher photon throughput, no thin film filters were employed. The unwanted spectral contributions were removed by the geometry of GIS and additional beam-stops inside the spectrometer housing.

The gas puff target approach allows one to change the working gas to illuminate the sample with different emission spectra, currently, the Kr gas was used with an energy range of 250–500 eV. However, other gasses can be used as well, to facilitate higher emission near other absorption edges of interest of different materials. 

Also, a novelty was to use a specially prepared gas mixture for easier (quicker) and more precise calibration of the spectrometer, since in one spectrum multiple transitions (spectral emission lines) from different gasses, present in the mixture, were visible and could be accounted for during the calibration process. 

Such a single-shot NEXAFS system could provide the possibility to perform test experiments on environmental, biological, and material science samples, to obtain preliminary data on novel materials and samples, without the immediate need to get beam time on the large-scale facility. Also, it may allow performing research on materials that may be too fragile or have other constraints and limitations that preclude measurements at a synchrotron source. This system may also be used for developing novel approaches to data processing with samples, which are later studied in more detail at large scale facilities. It also may, in the near future, allow for time-resolved studies and a broader spread of the NEXAFS spectroscopy to environmental, biological, and material sciences, which in turn, might benefit the development of these areas of science and technology.

## Figures and Tables

**Table 1:** Energy positions and assignments of features depicted in Figure 5, taken from [27], in the C-1s NEXAFS spectrum of PET foil, based on synchrotron data.

Peak Energy (eV)	Main Character	1s → (Orbital)	Area (%)	Analysis (Renormalized) (%)
284.4	ring	π*_C=C_	10.9	29.5
285.1	ring	π*_C=C_	8.7	23.6
287.0	carbohydrates	σ*_C–H_	1.4	3.8
287.8	carbonyl (C=O)	π*_C=O_	6.5	17.6
288.7	ring/C=O	π*_C=C_, π*_C=O_	8.1	22.0
289.8	ring	π*_C=C_	1.3	3.5
291.0	------ questionable -------		8.7	-
292.7		σ*	31.1	-
296.0		σ*	2.6	-
303.0		σ*	20.7	-

**Table 2:** The experimental elemental composition of the analyzed PET sample compared to the theoretical composition values. A 10% step function for each Gaussian type peak fitting curve was used as a fitting method.

Sample PET Foil 1 m Thick (C_10_H_8_O_4_)_n_	Method	Comment	Composition (%)			Global Error (%)
			C	H	O	
Theoretical	*w*/*w*%	calculated from the chemical formula	62.5	4.2	33.3	0
Experiment	10% step for each peak	NEXAFS spectrum from Figure 5	67.6	3.8	28.6	2.3
